# Prevalence of Latent Tuberculosis among Health Care Workers in High Burden Countries: A Systematic Review and Meta-Analysis

**DOI:** 10.1371/journal.pone.0164034

**Published:** 2016-10-06

**Authors:** Sharifa Nasreen, Mostafa Shokoohi, Monali S. Malvankar-Mehta

**Affiliations:** 1 Department of Epidemiology and Biostatistics, Schulich School of Medicine and Dentistry, Western University, London, Ontario, Canada; 2 HIV/STI Surveillance Research Center, and WHO Collaborating Center for HIV Surveillance, Institute for Futures Studies in Health, Kerman University of Medical Sciences, Kerman, Iran; 3 Department of Ophthalmology, Schulich School of Medicine and Dentistry, Western University, London, Ontario, Canada; University of Cape Town, SOUTH AFRICA

## Abstract

**Background:**

Tuberculosis is one of the leading causes of death worldwide. Twenty-two high burden countries contributed to the majority of worldwide tuberculosis cases in 2015. Health care workers are at high risk of acquiring tuberculosis through occupational exposure.

**Objective:**

To estimate the prevalence of latent tuberculosis infection (LTBI) among health care workers in high burden countries.

**Methods:**

Databases including MEDLINE (Ovid), EMBASE (Ovid), CINAHL (Ovid) and ISI Web of Science (Thompson-Reuters), and grey literature were searched for English language records on relevant medical subject headings (MeSH) terms of LTBI and health care providers. Literature was systematically reviewed using EPPI-Reviewer4 software. Prevalence and incidence of LTBI and 95% confidence intervals (CI) were reported. Pooled prevalence of LTBI and 95% CI were calculated using random-effects meta-analysis models and heterogeneity was assessed using *I*^*2*^ statistics. Sub-group analysis was conducted to assess the cause of heterogeneity.

**Results:**

A total of 990 records were identified. Of those, 18 studies from only 7 high burden countries representing 10,078 subjects were included. Tuberculin skin test results were available for 9,545 participants. The pooled prevalence of LTBI was 47% (95% CI 34% to 60%, *I*^*2*^ = 99.6%). In subgroup analyses according to the country of the study, the pooled prevalence of LTBI was lowest in Brazil (37%) and highest in South Africa (64%). The pooled prevalence of LTBI among medical and nursing students was 26% (95% CI 6% to 46%, *I*^*2*^ = 99.3%) while the prevalence among all types of health care workers was 57% (95% CI 44% to 70%, *I*^*2*^ = 99.1%). Incidence of LTBI was available for health care workers in four countries. The cumulative incidence ranged from 2.8% in Brazilian medical students to 38% among all types of health care workers in South Africa.

**Conclusion:**

The findings of this study suggest that there is a high burden of LTBI among health care workers in high burden countries. Adequate infection control measures are warranted to prevent and control transmission in health care settings.

## Introduction

Health care workers (HCWs) are at increased risk of acquiring tuberculosis (TB) than the general population [1]. The risk of TB in HCWs is of particular importance in the 22 high TB burden mostly low- and middle-income countries that account for 80% of global TB cases. Increased exposure and suboptimal infection control measures in high-burden countries increases the occupational risk of HCWs in resource-constrained settings [2–4]. Low background practice of cough etiquette and respiratory hygiene in general population can also contribute to transmission of respiratory infections including TB at physician-patient interface in high burden countries [5]. On the other hand, HCWs with active TB can be an important source of TB infection to others in both health care and community settings in addition to impeding health care delivery particularly in countries with suboptimal number of HCWs. The estimated annual incidence of active TB and latent TB infection (LTBI) was 2.4%–3.7% and 3.8%–8.4% respectively among HCWs with varying TB incidences in general population [1]

Latent tuberculosis infection (LTBI) does not produce disease manifestations and is not infectious albeit it results in persistent immune response against *Mycobacterium tuberculosis* antigens[6]. However, there remains a 10–15% life-time risk of developing active TB. While the majority of the cases develop within five years of infection, persons infected within previous two years are at high risk for progression to disease [7]. There is no gold standard test for diagnosis of LTBI. Tuberculin skin test (TST) and blood Interferon-gamma release assay (IGRA) tests are performed to diagnose LTBI. However, these tests are limited in their ability to distinguish latent infection from cured or treated infections and to predict progression to active tuberculosis [6]. Bacille Calmette-Guerin (BCG) vaccination and non-tuberculous mycobacteria can cause false positive TST results, thereby reducing the specificity of TST. Nevertheless, a systematic review on the effects of BCG vaccination and non-tuberculous mycobacteria found that BCG vaccine received in infancy does not affect TST particularly if the test is done more than 10 years after the vaccination. But BGC vaccination after infancy leads to frequent, persistent and larger TST reactions [8]. Since BCG vaccination in most of the high burden countries are given once in early infancy [9], it is likely that the vaccination would not affect TST undertaken later in life. On the other hand, despite higher specificity and less cross-reactivity due to BCG vaccination, frequent reversion and conversion has been reported from serial testing by IGRA rendering interpretation difficult [10, 11].

Several systematic reviews on tuberculosis among health care workers have been conducted previously [1, 3, 12–14]. The first review published more than 20 years back in 1995 included studies from five non-high burden countries the United States, Canada, United Kingdom, Japan and Hong Kong during 1934–1990 [12]. In 2005, a systematic review reporting frequency of a positive tuberculin test or tuberculin test conversion included studies from low incidence countries the United States, Spain, Canada, Germany, Australia and UK during 1971–99 [13]. Two systematic reviews published in 2006 and 2007 on prevalence of LTBI among health care workers in low, middle and high income countries included studies from four high burden countries Brazil, India, Uganda, and Thailand conducted during 1999–2005 [3, 14]. The latest systematic review and meta-analysis published in 2011 included studies from previous reviews and additional studies published during January 2005–July 2010 and reported incidence of LTBI in HCWs from studies conducted in only four high burden countries (Brazil, India, Zimbabwe and Thailand) during 1995–2005. This review excluded studies reporting only prevalence of LTBI and active TB among HCWs [1]. While national-level data on burden of tuberculosis in general population is available from, nationally representative data on LTBI burden in HCWs in the high burden countries is lacking. Data on HCW burden are available from individual studies alone. Since longitudinal studies are time consuming, labour intensive and expensive, prevalence studies are more likely to be conducted in resource-constrained settings and provide information on LTBI burden. A systematic review on current burden of LTBI among HCWs in high burden countries would provide evidence of existing occupational risk of infection in health care settings, raise awareness among HCWs to adopt and practice necessary infection control measures, and guide policy makers to explore and implement necessary prevention and control measures to reduce disease burden.

We performed a systematic review and meta-analysis to determine the prevalence and incidence of LTBI among HCWs in high burden countries by summarizing data identified in the published literature. We followed the MOOSE (Meta-Analyses and Systematic Reviews of Observational Studies) (S1 File) and the Preferred Items for Systematic Reviews and Meta-Analyses (PRISMA) (S2 File) guidelines for reporting of findings from this systematic review and meta-analysis [15, 16].

## Methods

### Search strategy

#### Database search

Four bibliographic databases were searched to identify studies on latent tuberculosis among health care workers: MEDLINE (Ovid), EMBASE (Ovid), CINAHL (Ovid) and ISI Web of Science (Thompson-Reuters). Initially two broad concepts ‘latent tuberculosis’ and ‘health care workers’ were used to identify the keywords for subject searching [for example, Medical Subject Headings (MeSH) in MEDLINE database] in the databases. The key word ‘Latent tuberculosis’ was used to search for the concept ‘latent tuberculosis’ with explosion feature. Several key words were used for the concept ‘health care workers’ such as ‘health care worker’, ‘health personnel’, ‘physicians’, ‘medical staff’, ‘hospital staff’, ‘nurses’, ‘community health worker’, ‘occupational exposure’, ‘nosocomial’, ‘cross infection’ and ‘hospital infection’. For each concept, both key words and subjects identified in the databases were used together with ‘OR’ for searching in each database. Then the keywords and subjects for each concept were combined with ‘AND’ to obtain final search result (Table 1 in S1 File).

#### Other sources

The index of the *International Journal of Tuberculosis and Lung Disease* was hand searched to identify additional articles not captured by the database search. The index of the journal *Tuberculosis* was also searched. Additionally, an article reporting the prevalence of LTBI among HCWs in Bangladesh and published in the *Health and Science Bulletin* (HSB) of icddr,b (formerly known as International Centre for Diarrhoeal Disease Research, Bangladesh), an international health research institute based in Bangladesh was included.

Grey literature was identified by searching for conference or meeting abstracts and proceedings, and dissertations. BIOSIS Previews at Web of Science interface was searched for conference or meeting abstracts or proceedings. Electronic Thesis Online Service (EThoS), Theses Canada Portal and the Networked Digital Library of Theses and Dissertations (NDLTD) were searched to identify dissertations on LTBI among HCWs. No record was identified in EThoS. Details of the search are provided in S3 File. Our protocol for the systematic review can be made available upon request.

### Inclusion and exclusion criteria

The following inclusion criteria were considered for the systematic review: studies published and conducted in any of the 22 high burden countries during 2001–2015, reporting prevalence of LTBI among health care facility workers or community health workers based on TST and published in English language. While the new interferon-gamma release assay (IGRA) is available and used in high and upper middle-income countries, the World Health Organization (WHO) strongly recommends TST for low and other middle-income countries [6]. Since the majority (17 out of 22) of the high burden countries belong to low and middle-income countries according to the World Bank income classification [17] diagnosis of LTBI based on TST was chosen for this review to maintain comparability of the prevalence from the studies.

Studies not conducted in high burden countries, not reporting prevalence of LTBI among HCWs, and diagnosis of LTBI based on IGRA alone were not included in the review. We also excluded previous review articles, letters to the editor, editorials and perspectives as they do not provide primary data.

### Screening

All identified records from different databases and additional records identified through grey literature search, and hand-searches of relevant journals were imported to EPPI-Reviewer4 (V.4.5.0.1) software to remove duplicates. After removal of duplicates, the records were screened by two reviewers (SN and MS) in three levels. The first level screening included title screening, the second level screening included abstract screening and the third level screening included full text screening. At each level, the reviewers separately screened the records and then merged their agreements and disagreements and calculated the kappa (κ) statistics. The inter-rater agreements (*Kappa* statistics) for the first, second and third-level of screening were 91% (0.86), 76% (0.63) and 87% (0.81) respectively. Disagreements were resolved by discussion before finalizing records for next level of screening. Differences between the reviewers were discussed and resolved by consensus. In cases where consensus was not achieved, a third reviewer was brought in to provide a decision. After screening, studies were assessed for eligibility and final selection.

### Study quality

Selected studies were assessed for quality using modified Joanna Briggs Institute Prevalence Critical Appraisal Tool [18] tailored to the objective and primary outcome measure of this review. The tool was modified to account for LTBI specific test criteria. Each study was assessed for eight criteria: representativeness of sample, participant recruitment, sample size, description of participants and setting, response rate, TST protocol, objective and reliable measurement of TST and BCG vaccination status (S4 File). Although 13 out of 18 studies met 6 or more criteria, none were excluded in meta-analysis based on the quality.

### Data extraction

Data was extracted using a data extraction MS Excel sheet. Data extraction included author, year of publication, study location, design, year of study, study settings, participants, number of study participants, age, number and percentage of females, PPD used, TST testing protocol, cut-off, reading interval, BCG scar or vaccination status, TB control measures at the health care setting, prevalence and incidence of LTBI. For studies that did not include mean (SD) age, median age was used as the mean age if sample size was >30. The available data on range, *p*-value, and confidence interval were utilized and converted to the common effect measure, SD.

SN conducted the search, data extraction, and analysis of study quality.

### Meta-analysis

Random effects model was used to estimate pooled prevalence across the studies assuming heterogeneity across the aggregated population using STATA version 13.0. A forest plot was generated to show prevalence estimates for individual studies. Heterogeneity was assessed by Pearson chi-square test and *I*^2^ test was performed to quantify the level of heterogeneity. An *I*^2^ score of >75% indicated high heterogeneity. Sub-group analysis was conducted for the country of study, TST protocol and type of HCW to explore the possible causes of heterogeneity. Forest plots were generated for each sub-group analysis. Funnel plot was generated to check publication bias using SAS version 9.4 software. The plot was generated by plotting the observed proportion against the number of TST participants and superimposing 95% (~2standard deviation) and 99.8% (~3 standard deviation) prediction limits around the overall proportion [19].

## Results

### Search results and study selection

**Fig 1:**
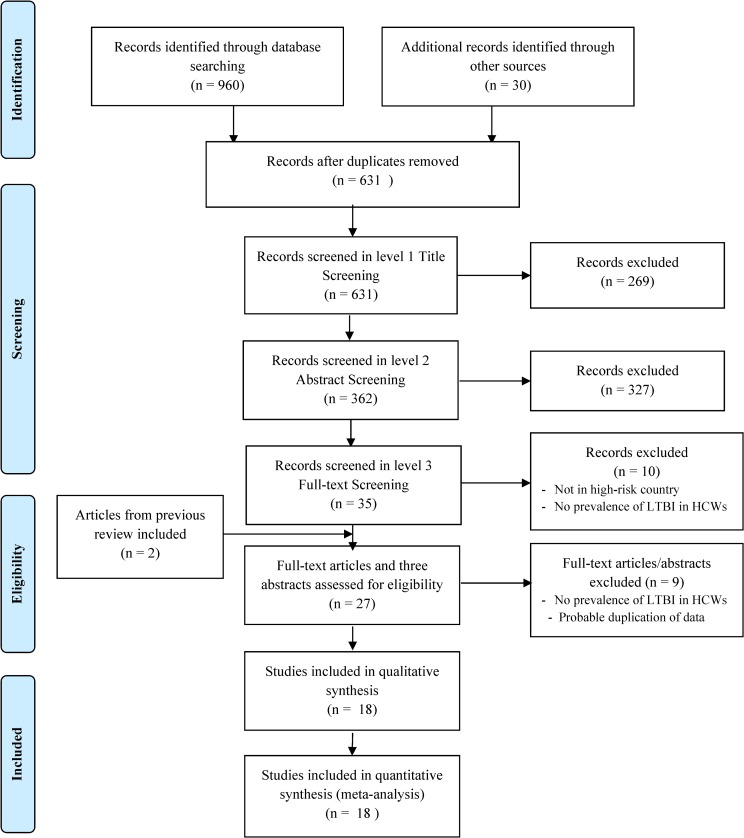
Flow diagram for selection of studies on latent tuberculosis infection among health care workers

A total of 990 records were identified from different databases, grey literature and relevant journals. Three-hundred and fifty-nine duplicate records were removed and remaining 631 records were screened by the 2 reviewers. After screening a total of 27 records including 22 research articles, three abstracts and two articles from previous reviews that did not come up in the search result were assessed for eligibility [20, 21]. Of these, an abstract of a full article in Portuguese language was in English and reported prevalence of LTBI among HCWs, and was included in both qualitative and quantitative synthesis [22]. Five articles and two abstracts were excluded for probable duplication [23–29]. One abstract that did not include the number of TST positives was excluded [30]. The corresponding author of another article was contacted to confirm duplication but did not respond and therefore was excluded [21]. In the end, a total of 18 studies were included for qualitative and quantitative synthesis (Fig 1).

### Study characteristics

Eighteen studies representing 10,078 subjects from seven high burden countries were included: one from Bangladesh [31], six from Brazil [22, 32–36], five from China [4, 37–40], two from India [41, 42], two from South Africa [43, 44], one from Uganda [45] and one from Zimbabwe [20] (Table 1). These studies were conducted during 2001–2014 and published during 2005–2015. One study from an abstract did not include the study time [22]. The study design included cross-sectional studies (eight studies) and prospective cohort studies (10 studies). Sample size ranged from 65 to 2,153.

**Table 1:** Characteristics of studies included in the systematic review and meta-analysis.

Author, year, and reference	Country	Study design	Year of study	Study setting	Study participants	No. of participants	Age	Age, Mean (SD)	Female, n/N (%)
Islam et al., 2014 [31]	Bangladesh	Cross-sectional	2013	Chest disease hospitals in 4 districts	Doctors, nurses, pharmacists, administrators, laboratory and support staff	482	Mean (range): 43.3 (18.2–65.6)	43 (10.37)	294/482 (61)
Rabahi et al., 2007 [32]	Brazil	Prospective cohort	cross-sectional 2001, cohort 2002–2004	A TB state reference centre	Healthcare workers	437	Mean (range): 40.9 (18–68)	40.9 (12.5)	345/413 (84)
Teixeira et al., 2011 [33]	Brazil	Prospective cohort	2002–2004	5 medical schools in low, intermediate and high TB incidence cities	Undergraduate medical students	1094	Mean (SD): TST positive (71): 22.0 (2.8); TST negative (961): 22.4 (3.1)	TST positive (71): 22.0 (2.8); TST negative (961): 22.4 (3.1)	532/1094 (49)
Severo et al., 2011 [34]	Brazil	Cross-sectional	2009–2010	A teaching hospital; Three wards where TB patients are treated including ICU	Nurses and nurse technicians	65	Mean (SD): 29.9 (6.7)	29.9 (6.7)	48/65 (87.3)
Miranda et al., 2012 [35]	Brazil	Prospective cohort	2006–2008	A clinic situated at the Federal University of Minas Gerais	Administrative staff, nursing professionals, physicians, psychologists, social assistants, pharmacists	251	Mean (range): 40 (17–69)	40 (13)	175/251 (69)
Rogerio et al., 2013 [22]	Brazil	Cross-sectional	not provided in the abstract	Federal University of Espirito Santo (UFES)	Medical and nursing students	225 (98 medical students, 127 nursing students)	not provided in the abstract		not provided in the abstract
de Souza et al., 2014 [36]	Brazil	Cross-sectional	2011–2012	4 cities with high incidence of TB	Primary HCWs: Physicians, nurses, nurse technicians and community health workers	664	Median (IQR): 42 (40.8–42.4)	42 (1.19)	565/664 (89)
He et al., 2010 [4]	China	Cross-sectional	2005	All 18 TB centers at 18 prefectures and 40 TB centers at the county/district level	Medical and administrative/logistic staff	2153	Mean (range): 37 (18–61)	37 (10.75)	1163/2153 (54)
Li-fan et al., 2013 [37]	China	Prospective cohort	2005–2011	A tertiary general hospital (medical college hospital)	Physicians, nurses, interns, laboratory staff	101	Median (IQR): 26 (22–32)	26 (7.41)	78/101 (77.2)
Wei et al., 2013 [38]	China	Prospective cohort	2009	Harbin Thoracic Hospital	Nurse, doctor, laboratory staff, administrator, other	210	Median (IQR): 31 (24–41)	31 (12.59)	163/210 (77.62)
Zhou et al., 2014 [39]	China	Cross-sectional	2011	2 public hospitals (Zhengzhou Central Hospital, Henan Provincial Infectious Disease Hospital)	Not specified but included HCWs from outpatient clinics, intensive care units, emergency, internal medicine, infectious disease, Chinese medicine, radiology, stomatology departments and laboratory	731	Mean (range): 31.4 (18–71)	31.4 (13.25)	188/529 (36)
He et al., 2015 [40]	China	Prospective cohort	2011–2012	2 counties	All licensed and registered village doctors	880	Median (range): 40 (19–77)	40 (14.5)	421/880 (47.8)
Pai et al., 2005 [41]	India	Cross-sectional	2004	A rural medical school	All healthcare workers: trainees, nurses, laboratory workers, orderlies, attending physicians	726	Median (range): 22 (18–61)	22 (10.75)	453/726 (62)
Christopher et al., 2014 [42]	India	Prospective cohort	2007–2009	A tertiary level referral medical school	Nursing students	800	Mean: 21.7		707/800 (94)
McCarthy et al., 2015 [43]	South Africa	Prospective cohort	2008–2009	3 public sector facilities	Nurses, counsellors, doctors and medical students	199 (120 HCWs, 79 medical students)	Median (IQR): HCWs 36 (28–46); medical students 22 (22–24)	HCWs: 36 (13.33); medical students 22 (1.48)	HCWs: 106/120 (88.3), medical students: 44/79 (55.7)
Adams et al., 2015 [44]	South Africa	Prospective cohort	May 2009-July 2011	5 community-based primary health care facilities and 2 secondary TB hospitals	Health work workers—support (administrative, security and lay healthcare workers) and clinical staff (interns, researchers, trainees, nurses, doctors) aged >18 years old	505	Not provided		407/505 (31)
Kayanja et al., 2005 [45]	Uganda	Cross-sectional	2001	3 hospitals: 1 national public referral hospital and 2 private hospitals	Doctors, nurses, techicians, support staff, students and others	396	Mean (SD): 31 (9.4))	31 (9.4)	268/396 (68)
Corbett et al., 2007 [20]	Zimbabwe	Prospective cohort	Followed-up upto 2005	Perirenyatwa and Harare Central Hospitals	nursing and polytechnic students	159 nursing students	Not provided		Not provided

### Publication bias

**Fig 2:**
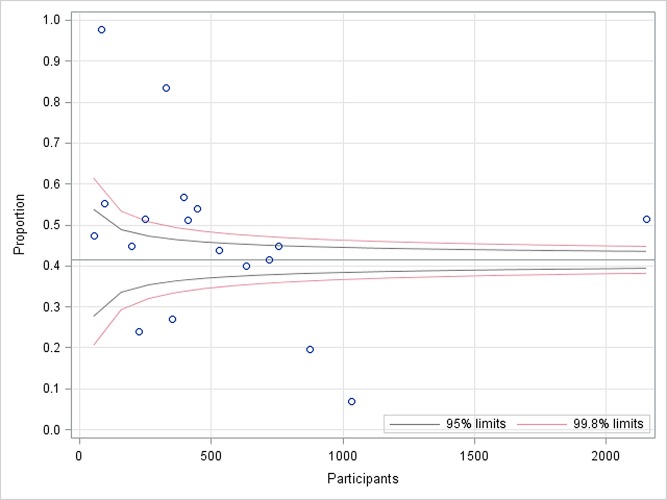
Funnel plot for included studies on latent tuberculosis among health care workers.

The funnel plot for the included studies was not symmetrical (Fig 2). Several of the included studies were outside the 99.8% limits. However, publication bias could not be concluded partially because of difficulty in interpretation of funnel plot for a group of studies, high heterogeneity and small effect sizes. Nevertheless, publication bias in only one of the several possible explanations for funnel plot asymmetry.

### Prevalence of LTBI

**Fig 3:**
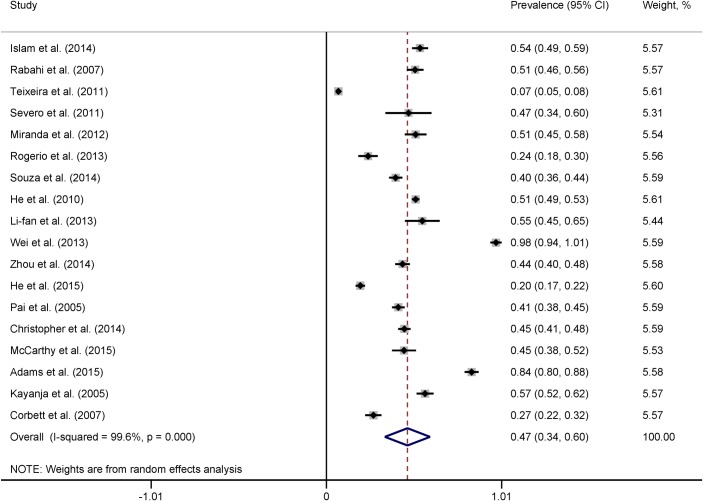
Forest plot showing study-specific and pooled estimates of prevalence of LTBI among health care workers.

LTBI was detected by two-step TST in six studies and one-step TST in the remaining 12 studies (Table 2). BCG scar or BGC vaccination at birth, infancy or childhood among the participants ranged from 32% to 98%. Infection control measures were suboptimal or virtually absent in six studies that provided information on TB control measures at the health-care settings [4, 32–34, 39, 41]. TST results were available for 9,545 HCWs and TST was positive among 3,951 of them. The prevalence of LTBI ranged from 6.9% among medical students to 97.6% among all types of HCWs. The pooled prevalence was 47% (95% CI 34% to 60%, *I*^2^ = 99.6%) (Fig 3). There was substantial heterogeneity across the studies and thus random-effect analysis was conducted.

**Table 2:** Prevalence of LTBI among health care workers.

Author, year, and reference	Country	Study participants	PPD used; TST Testing protocol; Cut off; Reading interval;	BCG scar or history of BCG vaccination, % (n/N)	Any TB control measures at the health-care setting	LTBI,n/N	Prevalence of LTBI (%, 95% CI)
Islam et al., 2014 [31]	Bangladesh	All types of HCWs	2 TU RT23 (0.1 mL); two-step; ≥10 mm; 48–72 h	82 (395/482)	-	242/449	54 (49.2 to 58.6)^*^
Rabahi et al., 2007 [32]	Brazil	All types of HCWs	2 TU RT23 (0.1 mL); two-step; ≥10 mm; 48–72 h	71 (293/413)	No HEPA filters in hospital rooms. HCWs did not use protective garments till the baseline study	211/413	51 (46.2 to 56.0)^*^
Teixeira et al., 2011 [33]	Brazil	Medical students	2 TU RT23 (0.1 mL); two-step; ≥10 mm; 48–72 h	83 (908/1094)	Students and patients used masks	71/1032	6.9 (5.4 to 8.6)
Severo et al., 2011 [34]	Brazil	Nursing professionals	TU? RT23 (0.1 mL); one-step; ≥10 mm; 48–72 h	98 (54/55)	Respiratory protection measures were not always taken	26/55	47.3 (33.7 to 61.2)^*^
Miranda et al., 2012 [35]	Brazil	All types of HCWs	2 TU RT23 (0.1 mL); two-step; >10 mm; 72–96 h	40 (101/251)	-	129/251	60 (45.0 to 57.7)^*^
Rogerio et al., 2013 [22]	Brazil	Medical and nursing students	one-step; ≥10 mm; 72 h	-	-	54/225	24 (18.6 to 30.1)^*^
de Souza et al., 2014 [36]	Brazil	All types of HCWs	2 TU RT23 (0.1 mL); one-step; ≥10 mm; 48–72 h	86 (546/632)	-	252/632	40 (36 to 43)^*^
He et al., 2010 [4]	China	All types of HCWs	5 TU RT23 (0.1 mL); one-step; ≥10 mm; 48–72 h	36 (777/2153)	No N95 masks for HCWs, no mechanical ventilation in the centers	1106/2153	51.4 (49.2 to 53.5)^*^
Li-fan et al., 2013 [37]	China	All types of HCWs	5 TU (0.1 mL); one-step; ≥10 mm; 72 h	98 (99/101)	-	53/96	55.2 (45.2 to 64.9)
Wei et al., 2013 [38]	China	All types of HCWs	5 TU (PPD); one-step; ≥10 mm; within 72 h	94 (198/210)	-	83/85	97.6 (91.8 to 99.7)^*^
Zhou et al., 2014 [39]	China	All types of HCWs	5 TU (PPD); one-step; ≥10 mm; 48–72 h	32 (167/528)	Masks including surgical mask and N95 respirator were used	232/529	43.9 (39.6 to 48.2)^*^
He et al., 2015 [40]	China	Village doctors	5 TU RT23 (0.1 mL); one-step; ≥10 mm; 48–72 h	36 (320/880)	-	171/875	19.5 (17.0 to 22.3)^*^
Pai et al., 2005 [41]	India	All types of HCWs	1 TU RT23; one-step; ≥10; 48–72 h	71 (514/726)	Limited infection control measures, no details	298/720	41 (38 to 45)
Christopher et al., 2014 [42]	India	Nursing students	2 TU RT23 (0.1 mL); two-step; ≥10 mm; 48–72 h	81 (613/755)	-	339/755	45 (41.3 to 48.5)^*^
McCarthy et al., 2015 [43]	South Africa	All types of HCWs and students	2 TU RT23 (0.1 mL); one-step; >10 mm; 48–72 h	-	-	89/199	44.7 (37.7 to 51.9)^*^
Adams et al., 2015 [44]	South Africa	All types of HCWs	2 TU RT23 (0.1 mL); one-step; ≥10 mm; 48–72 h	92 (313/505)	-	275/329	84 (79.1 to 87.4)^*^
Kayanja et al., 2005 [45]	Uganda	All types of HCWs and students	5 TU RT23 (0.1 mL); one-step; ≥10 mm; 72 h	66 (261/396)	-	225/396	57 (52 to 62)
Corbett et al., 2007 [20]	Zimbabwe	Nursing students	2 TU RT23 (0.1 mL); two-step; ≥10 mm; 48–72 h	Not specified	-	95/351	27.1 (22.5 to 32)^*^

*95% CI calculated using binomial distribution

**Fig 4:**
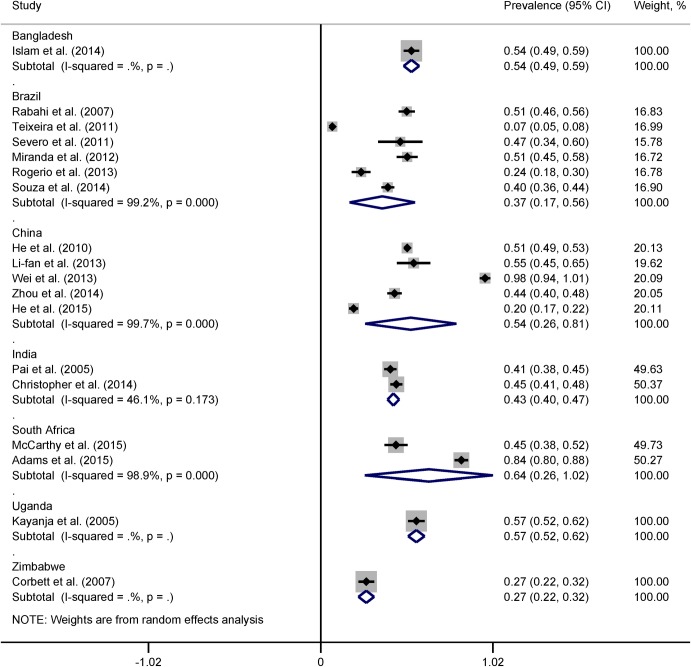
Forest plot showing country-specific pooled estimates of prevalence of LTBI.

**Fig 5:**
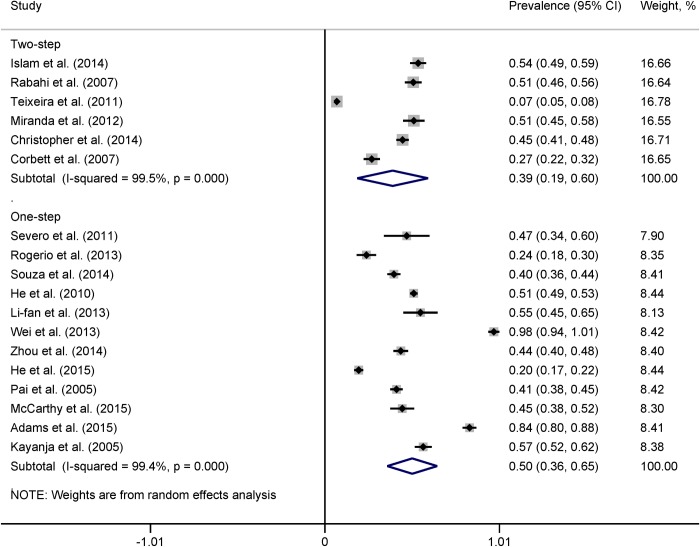
Forest plot showing pooled estimates of prevalence of LTBI according to two-step vs. one-step TST protocol.

**Fig 6:**
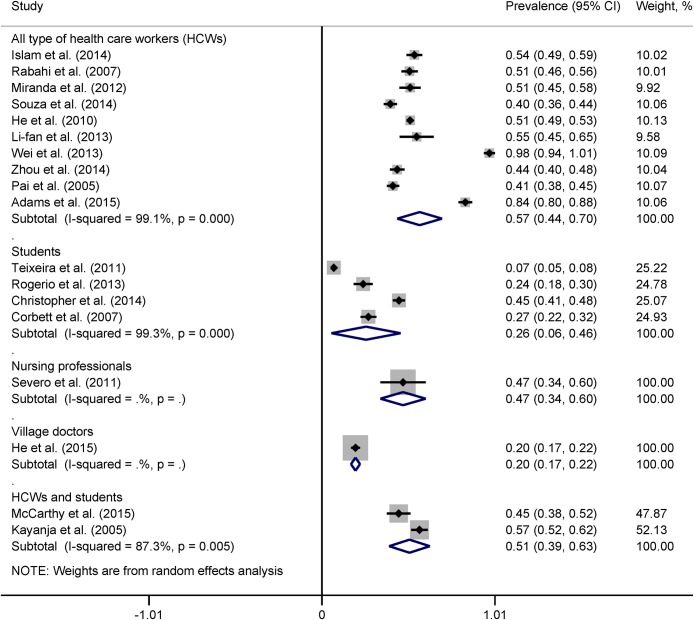
Forest plot showing pooled prevalence of LTBI according to health care worker type.

Heterogeneity across the studies remained in subgroup analyses. In country-wise subgroup analysis, the pooled prevalence of LTBI was lowest in Brazil (37%), followed by India (43%), China (54%), and South Africa (64%) among countries with more studies (Fig 4). However, heterogeneity between the two studies from India became statistically insignificant (*I*^2^ = 46.1%, p = 0.17). The pooled prevalence of LTBI was 39% (95% CI 19% to 60%, *I*^2^ = 99.5%) for studies with two-step TST and 50% (95% CI 36% to 65%, *I*^2^ = 99.4%) for studies with one-step TST protocol (Fig 5). The pooled prevalence of LTBI among medical and nursing students was 26% (95% CI 6% to 46%, *I*^2^ = 99.3%) while the prevalence among all types of HCWs was 57% (95% CI 44% to 70%, *I*^2^ = 99.1%) (Fig 6).

### Incidence of LTBI among health care workers

Incidence of LTBI was available for HCWs in Brazil, China, South Africa and Zimbabwe (Table 3). The cumulative incidence ranged from as low as 2.8% among Brazilian medical students to as high as 38% among all types of HCWs in South Africa. Nursing students in Zimbabwe had an incidence rate of 19.3/100 py (95% CI 14.2 to 26.2).

**Table 3:** Incidence of LTBI among health care workers.

Author, year, and reference	Country	Study participants	New LTBI, n/N	Cumulative Incidence of LTBI (95% CI)	Incidence rate per 100 py (95% CI)
Rabahi et al., 2007 [32]	Brazil	All types of health care workers	43/159	27% (19.6 to 36.4)^*^	-
Teixeira et al., 2011 [33]	Brazil	Medical students	13/458	2.8% (1.5 to 4.9)^*^	3.1 (1.8 to 5.2)
Miranda et al., 2012 [35]	Brazil	All types of health care workers	2/39	5.1% (0.62 to 18.5)^*^	-
He et al., 2015 [40]	China	Village doctors	53/465	11.4% (8.66 to 14.64)	11.4 (8.5 to 14.9)^*^
McCarthy et al., 2015 [43]	South Africa	Health care workers and students	25/93	27% (18 to 37)	29 (19 to 42)
Adams et al., 2015 [44]	South Africa	All types of health care workers	13/34	38% (22 to 56)	-
Corbett et al., 2007 [20]	Zimbabwe	Nursing students	-	-	19.3 (14.2 to 26.2)

^*^ 95% CI calculated using Poisson distribution

## Discussion

We identified 990 research articles, abstracts and theses after searching various bibliographic databases and grey literature. Eighteen studies with 10,078 participants from seven high burden countries were included for qualitative and quantitative synthesis.

Our results suggest that nearly half of the HCWs had LTBI. This is likely due to high exposure to TB patients in absence of optimum TB control measures in the high background TB prevalence in these countries. HCWs in Brazil had the lowest overall prevalence of LTBI. However, two [22, 33] out of six studies in Brazil were conducted among medical or nursing students, and one [33] of these two studies had a relatively low prevalence of LTBI which may have affected the overall prevalence. When prevalence of LTBI was stratified by the type of HCW, medical and nursing students in this review had 50% less LTBI infection rate compared to the rates from studies that included all types of HCWs, nursing professionals or both HCWs and students. The lower rates of LTBI among students are probably because of their differential exposure particularly shorter duration and less frequent exposure to TB patients compared to other HCWs.

We did not perform statistical test for funnel plot asymmetry. Different statistical tests such as those proposed by Begg and Mazumdar, Egger, Tang and Liu, Macaskill et al., Deeks et al., Harbord et al, Peters et al., Schwarzer et al., and Rücker et al. are more objective methods to determine the presence or absence of publication bias and to test funnel plot asymmetry in a meta-analysis [46]. However, these tests focus on publication bias in intervention studies or diagnostic test accuracy studies and test for asymmetry in conventionally constructed funnel plots for studies on intervention effect estimates. For example, Egger’s test is based on linear regression of odds ratio on its standard error, weighted by the inverse of the variance of the intervention effect estimate (log odds ratio). Study suggests that conventional funnel plots used for potential publication bias for meta-analysis of proportion studies are inaccurate [47]. Additionally, bias detection tests do not perform well when between-study heterogeneity is large [46, 48]. We generated funnel plot by plotting observed proportion against the number of TST participants and superimposing 95% and 99.8% prediction limits around the overall proportion. Hence, the commonly used tests will not be appropriate to test the funnel plot asymmetry for our study.

Given the high heterogeneity among the studies, a random effect model was applied to calculate the overall prevalence of LTBI. Subgroup analysis could not explain the cause of the heterogeneity across studies. However, the two studies from India had similar prevalence and heterogeneity became statistically insignificant although one study was conducted among nursing students employing two-step TST [24] while the other included all types of health care workers and performed one-step TST [41]. The high heterogeneity across all studies as well as among different studies conducted in a country (Brazil, China, and South Africa) is likely due to the differences in study settings, participants, methodological quality, exposure to TB patients, and presence and practice of control measures across the studies.

There are limitations of this study. In this review, we included studies published in English alone and reporting prevalence of LTBI based on TST according to the recommendation of the World Health Organization. Therefore, we may have missed studies from high TB burden countries that were published in languages other than English in national or local journals. However, if a journal was indexed in the databases we searched and the abstract of a study was available in English in the database, the study would have been identified during our search. Indeed, we included one study published in a journal in Portuguese and had the abstract in English was identified through our search [22]. Despite interpretational challenges, studies have reported both lower and higher prevalence of LTBI by IGRA compared to TST in TB high burden countries [38, 40]. As a result, our estimates of LTBI burden may be an under or overestimate of the actual burden among health care workers. However, a systematic review and meta-analysis of studies comparing performances of IGRA with TST suggested that there was no consistent differences in positive test results between the tests in high incidence settings [49].

Studies employing one-step or two-step TST to detect LTBI were included in this review and six of the studies employed two-step TST. If TST is performed years after infection with *Mycobacterium tuberculosis*, the ability to demonstrate immune reaction to tuberculin may wane over time and result in false-negative reaction at initial TST. A boosted reaction (positive reaction) may be generated during the repeat test in a two-step TST in these individuals indicating old TB infection. Studies reporting prevalence or incidence of LTBI based on one-step TST protocol may have been unable to detect old infections in individuals taking the TST for the first time. Although 11 studies reported that 66–98% of the participants received BCG vaccination, previous vaccination status is not expected to affect the TST results among the participants if the vaccine was administered after birth, during infancy or later but more than 10 years prior to the TST [8]. However, HCWs receiving BCG vaccination within 10 years prior to the TST or receiving a prior TST may have led to an overestimation of the prevalence or incidence of LTBI.

In conclusion, the findings from this study show that HCWs in high TB burden countries remain at increased risk of acquiring TB infection in spite of heterogeneity among the studies. Even with a conservative estimation that 10% of these HCWs will progress to active TB in their life time, this will have a significant impact on disease transmission as well as health care delivery in these countries. However, information on burden of LTBI among HCWs in the majority of the 22 high burden countries remains unknown. Studies on LTBI burden in HCWs in those countries would provide evidence base to consider necessary guidelines for TB prevention and control. Future studies should consider using uniform LTBI diagnostic protocols to generate comparable results across different countries. Appropriate infection control measures would be the key to prevent nosocomial transmission of TB among HCWs. Future research is needed to identify and test the effectiveness of feasible and affordable environmental control and respiratory protection measures in resource-constrained settings. HCWs in high burden countries could benefit from periodical screening for early detection of LTBI and monitoring the progression to active TB.

## Supporting Information

S1 File MOOSE Checklist. (DOC)

S2 File PRISMA 2009 Checklist. (DOC)

S3 File Details of database, journals and grey literature search. (DOC)

S4 File Study quality assessment. (DOC)
