# Supplementary material for: Prevalence of Latent Tuberculosis among Health Care Workers in High Burden Countries: A Systematic Review and Meta-Analysis
Source: PLoS One. 2016 Oct 6;11(10):e0164034. doi: 10.1371/journal.pone.0164034 (PMC5053544; doi:10.1371/journal.pone.0164034)
Supplement: S1 File — (DOC) [file pone.0164034.s001.doc]

**S1:** **MOOSE Checklist**

**Prevalence of latent tuberculosis among health care workers in high burden countries: a systematic review and meta-analysis**

| **Criteria** | | **Brief description of how the criteria were handled in the meta-analysis** |
| --- | --- | --- |
| **Reporting of background should include** | |  |
| √ | Problem definition | Health care workers are at increased risk of acquiring tuberculosis than the general population. The estimated annual incidence of latent tuberculosis infection (LTBI) among HCWs was between 3.8% and 8.4%. However, the recent burden of LTBI among health care workers in high TB burden countries yet need to be summarized quantitatively. |
|  | Hypothesis statement | Not applicable |
| √ | Description of study outcomes | Prevalence and incidence of LTBI |
| √ | Type of exposure or intervention used | Health care setting |
| √ | Type of study designs used | Cross-sectional and cohort studies |
| √ | Study population | Health care workers in high TB burden countries |
| **Reporting of search strategy should include** | |  |
| √ | Qualifications of searchers | The credentials of the two investigators SN and MS are indicated in the author list. |
| √ | Search strategy, including time period included in the synthesis and keywords | Time period for search: 2001 – October 2016. Details are provided in Supplemental information, S1: Table 1. |
| √ | Databases and registries searched | MEDLINE (Ovid), EMBASE (Ovid), CINAHL (Ovid), and ISI Web of Science (Thompson-Reuters) |
| √ | Search software used, name and version, including special features | We did not employ a search software. EPPI-Reviewer 4 (V.4.5.0.1) software used to compile records, eliminate duplications and screening. |
| √ | Use of hand searching | We hand searched the index of the *International Journal of Tuberculosis and Lung Disease* and the journal *Tuberculosis*. Grey literature was identified by searching BIOSIS Previews at Web of Science interface, Electronic Thesis Online Service (EThoS), Theses Canada Portal and the Networked Digital Library of Theses and Dissertations (NDLTD) |
| √ | List of citations located and those excluded, including justifications | Details of the literature search process are outlined in the flow chart (Figure 1). The citation list is available upon request. |
| √ | Method of addressing articles published in languages other than English | We included only the studies available (full-text or abstract) in English language. |
| √ | Method of handling abstracts and unpublished studies | We included one abstract in English for a study published in Portuguese language. |
| √ | Description of any contact with authors | We contacted the corresponding author of one article to confirm duplication but did not receive any response. |
| **Reporting of methods should include** | |  |
| √ | Description of relevance or appropriateness of studies assembled for assessing the hypothesis to be tested | Detailed inclusion and exclusion criteria are described in the methods section. |
| √ | Rationale for the selection and coding of data | Data extracted from each of the studies were relevant to the population characteristics, study design, exposure, and outcome. |
|  | Assessment of confounding | Not applicable |
| √ | Assessment of study quality, including blinding of quality assessors; stratification or regression on possible predictors of study results | We assessed the quality of the studies using modified Joanna Briggs Institute Prevalence Critical Appraisal Tool tailored to the objective and primary outcome measure of this study. Details are provided in the methods section and supplemental information, S2: Table 2. |
| √ | Assessment of heterogeneity | Heterogeneity was assessed by Pearson chi-square test and *I*^2^ test was performed to quantify the level of heterogeneity. Subgroup analysis was also performed to explore possible causes of heterogeneity. |
| √ | Description of statistical methods in sufficient detail to be replicated | Description of methods of meta-analyses, and assessment of publication bias are detailed in the methods. |
| √ | Provision of appropriate tables and graphics | We included 1 flow chart,1 study characteristics table, 1 table on prevalence, 1 table on incidence, forest plots of all studies, and for sub-group analysis, and 1 funnel plot We have also included details of database search as supplementary information. |
| **Reporting of results should include** | |  |
| √ | Graph summarizing individual study estimates and overall estimate | Figure 3a |
| √ | Table giving descriptive information for each study included | Table 1 |
| √ | Results of sensitivity testing | Results of sub-group analysis: Figures 3b-3d |
| √ | Indication of statistical uncertainty of findings | 95% confidence intervals were presented with all summary estimates |
| **Reporting of discussion should include** | |  |
| √ | Quantitative assessment of bias | We assessed the quality of the studies using modified Joanna Briggs Institute Prevalence Critical Appraisal Tool tailored to the objective and primary outcome measure of this study. Details are provided in the methods section and supplemental information, S2: Table 2 for details. |
| √ | Justification for exclusion | We excluded studies where diagnosis of LTBI was based on Interferon-gamma release assay alone, and studies published in languages other than English. |
| √ | Assessment of quality of included studies | We discussed the results of the sub-group analyses, and potential reasons for the observed heterogeneity. |
| **Reporting of conclusions should include** | |  |
|  | Consideration of alternative explanations for observed results | Not applicable. |
| √ | Generalization of the conclusions | Our findings suggest that health care workers in high TB burden countries remain at risk of acquiring TB infection. We noted the lack of LTBI burden information from the majority of the high TB burden countries. |
| √ | Guidelines for future research | We recommend future studies on the burden of LTBI among health care works in TB high burden countries, preferably using uniform LTBI diagnostic protocols for comparable results. We also recommended future research to identify and test the effectiveness of feasible and affordable measures for prevention and control in resource-constraint settings. |
| √ | Disclosure of funding source | No funding was necessary for the undertaking of this systematic review. |
