# Supplementary material for: Prevalence of Latent Tuberculosis among Health Care Workers in High Burden Countries: A Systematic Review and Meta-Analysis
Source: PLoS One. 2016 Oct 6;11(10):e0164034. doi: 10.1371/journal.pone.0164034 (PMC5053544; doi:10.1371/journal.pone.0164034)
Supplement: S3 File — (DOC) [file pone.0164034.s003.doc]

**S3**: Details of database, journals and grey literature search

| **Database** | **Search words** | **Date of search** | **Number of records** |
| --- | --- | --- | --- |
| **MEDLINE (Ovid)** | (Latent tuberculosis.mp. or exp Latent Tuberculosis/) AND (Health Personnel/ or Occupational Exposure/ or Health care worker.mp. or Cross Infection/ OR Physician.mp. or Physicians/ OR Personnel, Hospital/ or Nursing Staff, Hospital/ or Hospital staff.mp. OR Nurses.mp. or Nurses/ OR Community health workers.mp. or Community Health Workers/) | 6 October 2015 | 201 |
| **EMBASE (Ovid)** | (Latent tuberculosis.mp. or exp Latent Tuberculosis/) AND (Health care worker.mp. or health care personnel/ OR physician/ or Physician.mp. OR Hospital staff.mp. or hospital personnel/ OR cross infection/ or hospital infection/ or nosocomial.mp. OR Nurses.mp. or nurse/ OR Community health workers.mp. or health auxiliary/) | 6 October 2015 | 432 |
| **CINAHL (Ovid)** | [(“latent tuberculosis” )] AND [(MH “Health Personnel”) OR [(MH “Rural Health Personnel”) OR “Health care worker”] OR [“Physician”] OR [(MH “Nursing Staff, Hospital”) OR (MH “Medical Staff, Hospital”) OR (MH “Personnel, Health Facility”) OR (MH “Medical Staff”) OR “Hospital staff”] OR [(MH “Cross Infection”) OR “Nosocomial” ] OR [(MH “Nurses”) OR “Nurses”] OR (MH “Community Health Workers”) OR “Community health workers”] | 6 October 2015 | 41 |
| **ISI Web of Science (Thompson- Reuters)** | TOPIC: (Latent tuberculosis) AND TOPIC: (Health care workers) | 6 October 2015 | 286 |
| **International Journal of Tuberculosis and lung disease** | Latent tuberculosis AND health care workers | 6 October 2015 | 11 |
| **Tuberculosis** | Latent tuberculosis AND health care workers | 14 October 2015 | 9 |
| **BIOSIS Previews** | Latent tuberculosis AND health care workers (Year 2001-2015, meetings) | 6 October 2015 | 6 |
| **Theses Canada Portal** | Latent tuberculosis AND health care workers | 11 October 2015 | 1 |
| **Networked Digital Library of Theses and Dissertations (NDLTD)** | Latent tuberculosis AND health care workers AND Tuberculin skin test (language: English) | 11 October 2015 | 2 |
| **Total** |  |  | **989** |

## 
