# Supplementary material for: Prevalence of Latent Tuberculosis among Health Care Workers in High Burden Countries: A Systematic Review and Meta-Analysis
Source: PLoS One. 2016 Oct 6;11(10):e0164034. doi: 10.1371/journal.pone.0164034 (PMC5053544; doi:10.1371/journal.pone.0164034)
Supplement: S4 File — (DOC) [file pone.0164034.s004.doc]

## S4: Study quality assessment

| **Author, year, and reference** | **Representativeness of sample** | **Participant recruitment** | **Sample size^*^** | **Description of participants and setting** | **Response rate, %^†^** | **TST protocol** | **Objective, reliable measurement of TST** | **BCG vaccination status provided** | **Quality indicators met** |
| --- | --- | --- | --- | --- | --- | --- | --- | --- | --- |
| Islam et al., 2014 [31] | Yes | Invited all eligible persons | Adequate | Adequate | High | Two-step | Yes | Yes | 8 |
| Rabahi et al., 2007 [32] | Yes | Invited all eligible persons | Adequate | Inadequate | High | Two-step | Yes | Yes | 7 |
| Teixeira et al., 2011 [33] | Yes | Unclear | Adequate | Adequate | High | Two-step | Yes | Yes | 7 |
| Severo et al., 2011 [34] | Yes | Invited all eligible persons | Inadequate | Adequate | High | One-step | Yes | Yes | 6 |
| Miranda et al., 2012 [35] | Yes | Invited all eligible persons | Adequate | Adequate | High | Two-step | Yes | Yes | 8 |
| Rogerio et al., 2013 [22] | Yes | Not provided in the abstract | Adequate | Adequate | Not provided | One-step | Yes | No | 4 |
| de Souza et al., 2014 [36] | Yes | Unclear | Adequate | Adequate | Not provided | One-step | Yes | Yes | 5 |
| He et al., 2010 [4] | Yes | Invited all eligible persons | Adequate | Adequate | High | One-step | Yes | Yes | 7 |
| Li-fan et al., 2013 [37] | Yes | Invited all eligible persons | Inadequate | Adequate | High | One-step | Yes | Yes | 6 |
| Wei et al., 2013 [38] | Yes | Unclear | Adequate | Adequate | Not provided | One-step | Yes | Yes | 5 |
| Zhou et al., 2014 [39] | Yes | Random | Adequate | Inadequate | High | One-step | Yes | Yes | 6 |
| He et al., 2015 [40] | Yes | Invited all eligible persons | Adequate | Adequate | High | One-step | Yes | Yes | 7 |
| Pai et al., 2005 [41] | Yes | Invited all eligible persons | Adequate | Adequate | Intermediate | One-step | Yes | Yes | 6.5 |
| Christopher et al., 2014 [42] | Yes | Invited all eligible persons | Adequate | Adequate | Not provided | Two-step | Yes | Yes | 7 |
| McCarthy et al., 2015 [43] | Yes | Invited all eligible persons | Inadequate | Adequate | Low | One-step | Yes | No | 4 |
| Adams et al., 2015 [44] | Yes | Unclear | Adequate | Adequate | Intermediate | One-step | Yes | Yes | 5.5 |
| Kayanja et al., 2005 [45] | Yes | Unclear | Adequate | Adequate | High | One-step | Yes | Yes | 6 |
| Corbett et al., 2007 [20] | Yes | Invited all eligible persons | Inadequate | Adequate | High | Two-step | Yes | No | 6 |

^*^ A sample size of ≥200 was considered as adequate and a sample size of <200 was considered as inadequate

^†^ A response rate of <50% was considered as low=0, between 50-80% was considered as intermediate=0.5 and >80% was considered as high=1
